# Ophthalmic manifestations as the first presenting feature in dengue fever: a 10-year study

**DOI:** 10.22336/rjo.2024.07

**Published:** 2024

**Authors:** Avinash Mishra, Anchal Tripathi, Atul Bhirud, Mohini Agrawal, Sandeep Gupta, JKS Parihar

**Affiliations:** * Department of Ophthalmology, Military Hospital, Jalandhar, Punjab, India; ** Department of Ophthalmology, Military Hospital, Jammu, India; *** Department of Ophthalmology, Command Hospital, Chandimandir, Punjab, India; **** Department of Ophthalmology, Centre for Sight, New Delhi, India

**Keywords:** ophthalmic manifestations, dengue, first presentation, fever

## Abstract

**Purpose:** To report patients who first presented with various ocular manifestations and eventually ascertained to have underlying dengue.

**Methods:** A prospective study was conducted at multiple tertiary eye-care centers in India from 2012 to 2022. Cases reporting initially with ocular features along with fever/past history of fever over the last two weeks or with clinical features of dengue were selected. After an ophthalmological examination, patients underwent complete serological and biochemical analysis and those with reduced platelet counts were evaluated for dengue.

**Results:** Out of 564 cases, 15 patients were verified to be afflicted with dengue eventually. A rising trend of cases was seen every year and out of 15 cases, eight cases were reported during the Covid-19 pandemic (from 2020 to 2022), but were COVID-negative. 9 cases presented with acute redness followed by diminished vision. Seven cases presented a history of fever over the last few days and one had traveled from dengue endemic area. The various ocular presentations included subconjunctival hemorrhage, viral keratitis, anterior uveitis, sixth-nerve palsy, and vitreous hemorrhage. On serological examination, all 15 patients were detected to have low platelets. All cases responded well with supportive treatment and the ocular features subsided in all within a couple of weeks with good visual recovery.

**Conclusion:** In a tropical nation, such as India, with endemic dengue zones and increasing figures of dengue lately, ophthalmologists must include dengue fever among the differential diagnoses in various ocular presentations like subconjunctival hemorrhage, viral keratitis, anterior uveitis, sixth nerve palsy, and vitreous hemorrhage.

**Abbreviations:** DHF = dengue hemorrhagic fever, PCR = polymerase chain reaction, RT-PCR = real-time automated reverse transcriptase (RT-PCR), SD = standard deviation, MAC-ELIS = IgM antibodies capture enzyme-linked immunosorbent assay, RE = right eye, LE = left eye, CECT = Contrast-enhanced computed tomography

## Introduction

Dengue fever results from infection via a flavivirus, spread by Aedes mosquitoes, with global prevalence [**1**]. Fever is among its numerous and varied presentations. Its progression to dengue hemorrhagic fever (DHF) demands urgent management, which, if left untreated, might result in lethal complications. With growing vigilance, less explored atypical presentations including neurological and ophthalmological involvement are gaining more recognition and are now classified as Dengue Expanded Syndrome [**2**].

Ophthalmic symptoms emerge in the form of anterior uveitis, pan-uveitis, retinitis, retinal hemorrhages, maculopathy, optic neuropathy, and corneal epitheliopathy [**3**-**5**]. Over the last few years, intense versions of ocular and orbital manifestations have additionally been documented, like necrotizing scleritis and panophthalmitis [**6**-**9**]. 

However, the present literature is limited to case reports. Moreover, to the finest of our understanding, there has been no literature to date on ocular features as the first and only presenting feature, which finally resulted in identifying dengue fever as the, in fact, causative etiology. Here, in the present study, we reported 15 cases that first presented to our ophthalmology department with various ocular clinical features, and which were eventually uncovered to have been caused because of an underlying dengue fever.

## Methods

A multicentric, prospective study was carried out in the department of Ophthalmology at multiple tertiary eye-care centers in North-Eastern India, from January 2012 to December 2022. It was authorized by the Institutional Ethics Committee of the respective institutions and was performed in compliance with the Declaration of Helsinki. Written informed consent was procured from all participants.

All cases presenting with ocular features along with fever, or previous history of fever in the last two weeks, or typical clinical manifestations of dengue, were selected for our study. A full ophthalmological examination was conducted for all patients, encompassing a complete slit-lamp anterior segment examination, in addition to, a dilated fundus examination with a 90D lens. Ophthalmic investigations (for example, optical coherence tomography, and fundus fluorescein angiography) were performed for posterior segment diseases, as required. Patients with a history of ocular trauma, past ocular surgery, long-term medications (such as anti-platelets), or any prior ocular morbidity like chronic ocular surface, inflammation, retinal diseases, etc. were not included in the study. 

564 cases met our inclusion criteria. After a detailed ophthalmological examination, patients underwent complete serological and biochemical tests. Patients, who had platelet count below 1.5 lakhs/cubic mm of blood, were further evaluated for dengue fever. Out of these, 15 cases were diagnosed with dengue fever. 

The identification of dengue fever was further confirmed by a referring internist, derived from distinctive history or clinical features (fever, recovering stage from fever, history of travel to dengue endemic area) and confirmed either by dengue polymerase chain reaction (PCR) and/or dengue serology (IgM and IgG seroconversion). The real-time automated reverse transcriptase (RT-PCR) assay was done with the Dengue LC RealArtTM RT-PCR Kit, in patients having a fever of less than five days. In patients suffering from pyrexia, for more than five days, serology studies were performed with the PanBioTM Dengue Duo IgM and IgG Rapid Strip Test. All the patients were administered conservatively, through the involvement of both an ophthalmologist and a physician.

**Statistical analysis**

Statistical analysis was accomplished using SPSS software version 25. Data was entered in numbers with descriptive data presented as mean ± standard deviation (SD), median, range, and percentage. The confidence interval was maintained at 95%, and a p-value of <0.05 was regarded as statistically significant.

## Results

Five hundred and sixty-four patients (564) met the inclusion criteria in our study of 10 years, out of whom, 15 patients were confirmed as a case of dengue fever. Out of this group, nine (60%) were males and six (40%) were females with an age group ranging between 20 to 69 years. The mean age at onset was 32.62±11 years. **Table 1** represents the first presenting ocular features, until then, undiagnosed instances of dengue fever. An upward trajectory of cases was seen annually, and out of the 15 cases, eight cases presented during the Covid-19 pandemic years, that is, between the years 2020 to 2022, but were found to be COVID-negative.

Most of the patients (9 cases) presented with ocular complaints of acute redness of the eye, followed by diminution of vision. Many of them (7 cases) had a history of fever for the past few days and one of them traveled from a dengue-endemic area. 

**Table 1:** Distribution of patients and demographic attributes based on the first presenting ocular signs until then, in undiagnosed cases of dengue fever

Ocular signs	Number of patients (n)	Age (years)	Comorbidities/ history of travel to endemic area	First ocular complaint	Best-corrected visual acuity	Reason for referral to medical OPD	Diagnosed during the Covid pandemic
Subconjunctival hemorrhage	8	21-43	Nil	Redness	6/6	3/8 had fever, 2/8 recovered from fever, 3/8 referred for blood pressure review	4/8
Viral keratoconjunctivitis	1	28	Travel to dengue endemic area	Redness	6/9	Recovered from fever	1/1
Anterior uveitis	1	32	Nil	Diminution of vision	6/12	Fever for 2 days	No
Sixth nerve palsy	2	28 and 35	Nil	Deviation of eye	6/9	Referred to rule out medical causes for neuropathy	1/2
Vitreous hemorrhage	3	27, 38, 40	Nil	Diminution of vision and floaters	3/60-6/60	Fever and myalgia	2/3

Patients were referred to the medicine department for further investigations in case they had a fever or were recovering from the same. 3 out of 8 patients with subconjunctival hemorrhage were referred to rule out medical causes like hypertension, as there was no comorbidity in them. In the case of sixth nerve palsy, the patients were evaluated to rule out medical and surgical causes for the neuropathy. 

On serological examination, all 15 patients were found to have low-to-borderline platelets value and were investigated for dengue with dengue IgM antibodies capture enzyme-linked immunosorbent assay (MAC-ELISA) test that confirmed the diagnosis. On admission, their pulse ranged between 90-96/min regular and blood pressure ranged between 140-90-106/70 mm of Hg with no significant postural fall with low to high-grade fever. The random blood sugar levels were normal in all these cases. A hemogram revealed leucopenia and a platelet count from 60,000/mm3 to 93,000/mm3. Liver enzymes and renal functions were normal. The chest radiographs were normal. They were managed with intravenous fluids according to 2009 WHO dengue guidelines. Contrast-enhanced computed tomography (CECT) of the brain was performed and reported as normal.

**Fig. 1:**
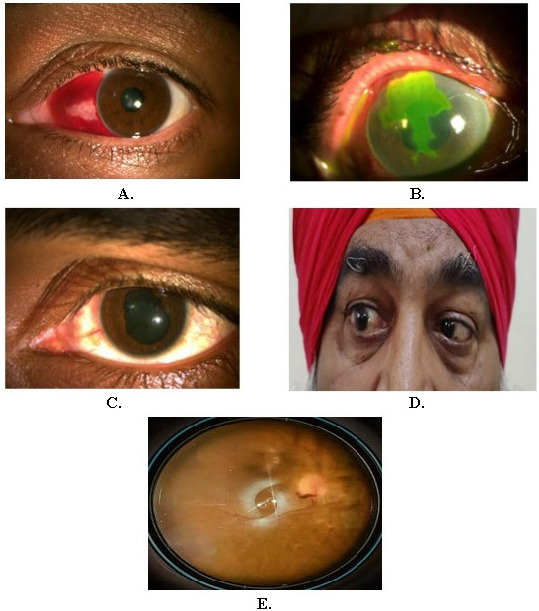
First presentation with ocular signs in, then, undiagnosed cases of dengue fever (**A**) subconjunctival hemorrhage; (**B**) viral keratoconjunctivitis; (**C**) acute anterior uveitis; (**D**) sixth-nerve palsy; and (**E**) small vitreous hemorrhage

All the cases responded well to the supportive treatment. No patient had to undergo any surgical intervention. The case of viral keratoconjunctivitis resolved after the use of topical antivirals and the acute anterior uveitis case required topical steroids. Both cases had platelet levels between 68,000 to 72,000/mm3. No further complication occurred and the ocular features subsided in all the patients within a week with good visual recovery. **Fig. 1 A-E** shows various first presentations with ophthalmic manifestations, then undiagnosed, in occurrences of dengue fever like (**A**) subconjunctival hemorrhage, (**B**) viral keratoconjunctivitis, (**C**) acute anterior uveitis, (**D**) sixth-nerve palsy and (**E**) small vitreous hemorrhage.

## Discussion

Ocular features in dengue fever are a rarely investigated aspect of dengue expanded syndrome. Ocular complications, although earlier thought to be uncommon, are now increasingly reported every year [**9**]. 

Reported sequelae of dengue fever in the eye include conjunctival petechial hemorrhages (commonest), anterior uveitis, vitreous hemorrhage, retinal hemorrhages, cotton-wool spots, disc edema and maculopathy [**9**-**12**]. Platelet counts less than 50,000/mL have been seen to predispose to ocular hemorrhages [**13**]. In contrast to this, our study showed subconjunctival hemorrhage as the most common first presenting feature in otherwise undiagnosed patients of dengue, with platelet count >50,000/ml. 

The anterior segment manifestations have been recorded in the form of subconjunctival hemorrhage and anterior uveitis, in certain previous studies [**14**], but the reported cases manifested these ocular features after 15 days of fever and platelets at the level of 9,000/µL. Whereas, in our study, there was a current history of fever and the platelet levels were >60,000/µl.

Bawankar et al. reported a case of stromal keratitis in dengue fever, in a 25-year-old female [**15**]. The patient developed stromal keratitis 15 days post dengue fever. However, in our research, the patient presented with viral keratoconjunctivitis first, and was later diagnosed as dengue positive, on further investigation, since the patient had traveled from a dengue-endemic zone. There have been case reports of acute viral conjunctivitis in patients suffering from dengue fever [**16**]. The average duration of the presentation ranged from one day to one week. Our patient was managed with topical antibiotics and lubricants, along with mild topical steroids. The patient improved satisfactorily with the ocular treatment and was completely asymptomatic within a week. 

Nainiwal et al. reported a case of a young female presenting with bilateral mild vitreous hemorrhage after 7 days of dengue fever [**17**]. Vitreous hemorrhage is also reported in several other studies [**18**-**20**]. The timeframe of presentation in all those cases was above 20 days after being diagnosed with dengue fever. Whereas, in this study, all the patients with dengue-related vitreous hemorrhage, presented with blurring of vision first. On further evaluation for defective vision, they were found to have vitreous hemorrhage. While investigating the etiology of vitreous hemorrhage in all these patients, the platelet count was observed to be <70,000/µl. The patients were directed to the medicine department for further evaluation, where they were diagnosed with dengue fever. All such patients were started on oral steroids, in consultation with the medical specialist, and were advised head end elevation. 

Dengue-associated cranial neuropathies, especially involving the abducens nerve, are relatively rare, with only extremely few reported cases [**10**,**11**]. To our surprise, our study reported two cases of abducens nerve cranial neuropathy as the first presenting feature until then, in undiagnosed cases of dengue. Thus, an elevated level of suspicion is essential while evaluating cases of cranial neuropathies in adults; a diagnosis of dengue should be kept in mind, particularly in cases coming from dengue endemic zones. In our study, a thorough history taking aided and guided us to the final diagnosis of the patient. Though unknown, the pathogenesis for neuro-ophthalmic complications is considered to be immune-mediated [**12**].

This study also underlined the circumstance that dengue-related ocular pathologies, such as conjunctivitis and sub-conjunctival hemorrhage, are frequently a self-limiting entity and resolve spontaneously without the need for any treatment, with good visual outcomes. However, prompt management is required in cases, such as uveitis, and posterior segment manifestations. Topical/oral steroids can be given, based on ocular involvement. For instance, in our case, topical prednisolone did wonders in patients with dengue-associated acute uveitis. The visual outcome of dengue ophthalmic complications is favorable if diagnosed and managed on time. Otherwise, a few patients may demonstrate persistent scotomas regardless of the clinical resolution of dengue ocular complications. 

Nonetheless, a significant number of cases with ophthalmic manifestations of dengue fever were recorded in the previous three years of our study, the explanation for this might be the global pandemic of COVID-19 as a chief cause of fever throughout this duration, leading the patients to perform self-testing for COVID-19 using available self-testing home kits. If negative, patients must have been refraining from reporting any fever to the hospital due to the prevalent social stigma attached to fever during those times. This might cause a high number of cases presenting to the ophthalmology OPD first with ocular features. The additional causes of fever, like dengue, have taken a back seat on account of the COVID-19 pandemic, as was observed in our study. Thus, a lack of awareness and concern over the non-reporting of the fever cases might have resulted in ocular morbidity due to dengue fever. 

The mechanism for dengue-linked ocular complications remains unidentified, yet conjectured to be associated with some immune-mediated mechanism and most likely with dengue serotyping. Further research is needed to explain the same. Ophthalmologists and physicians should be cognizant enough regarding the ocular features of dengue, and it is crucial to keep dengue fever as one of the differential diagnoses in similar instances.

## Conclusion

Thus, to conclude, this study described various ocular features as presenting features of dengue fever. It is recommended that in a tropical state, like India, with native dengue zones and increasing figures of dengue cases lately, ophthalmologists must keep dengue fever as a part of the differential diagnoses in various ocular presentations like subconjunctival hemorrhage, viral keratitis, anterior uveitis, sixth nerve palsy, and vitreous hemorrhage.

**Conflict of Interest Statement**

The authors declare no conflict of interest.

**Informed Consent and Human and Animal Rights Statement**

Informed written consent was obtained from all the patients.

**Authorization for the use of human subjects**

Ethical approval: The research related to human use complied with all relevant national regulations and institutional policies, followed the tenets of the Helsinki Declaration, and was approved by the Institutional Ethics Committee of the Department of Ophthalmology, Military Hospital, Jalandhar, Punjab, India (01/06/MAY/MH/JRC/2023 dt 23/05/2023).

**Acknowledgments**

Nil.

**Sources of Funding**

Nil. 

**Disclosures**

Nil.

**Presentation at a meeting**

NA.
